# Virus-host relationships of marine single-celled eukaryotes resolved from metatranscriptomics

**DOI:** 10.1038/ncomms16054

**Published:** 2017-06-28

**Authors:** Mohammad Moniruzzaman, Louie L. Wurch, Harriet Alexander, Sonya T. Dyhrman, Christopher J. Gobler, Steven W. Wilhelm

**Affiliations:** 1 Department of Microbiology, The University of Tennessee, Knoxville, Tennessee 37996, USA; 2 Department of Biology, James Madison University, Harrisonburg, Virginia 22807, USA; 3 Department of Earth and Environmental Science and Lamont-Doherty Earth Observatory, Columbia University, Palisades, New York 10964, USA; 4 School of Marine and Atmospheric Sciences, Stony Brook University, Stony Brook, New York 11794, USA

## Abstract

Establishing virus–host relationships has historically relied on culture-dependent approaches. Here we report on the use of marine metatranscriptomics to probe virus–host relationships. Statistical co-occurrence analyses of dsDNA, ssRNA and dsRNA viral markers of polyadenylation-selected RNA sequences from microbial communities dominated by *Aureococcus anophagefferens* (Quantuck Bay, NY), and diatoms (Narragansett Bay, RI) show active infections by diverse giant viruses (NCLDVs) associated with algal and nonalgal hosts. Ongoing infections of *A. anophagefferens* by a known *Mimiviridae* (AaV) occur during bloom peak and decline. Bloom decline is also accompanied by increased activity of viruses other than AaV, including (+) ssRNA viruses. In Narragansett Bay, increased temporal resolution reveals active NCLDVs with both ‘boom-and-bust’ and ‘steady-state infection’-like ecologies that include known as well as novel virus–host interactions. Our approach offers a method for screening active viral infections and develops links between viruses and their potential hosts *in situ*. Our observations further demonstrate that previously unknown virus–host relationships in marine systems are abundant.

## Abstract (web summary)

Viruses are partners in ecosystem ecology, yet their study has been primarily limited to laboratory models virus-host or derived from metagenomics. Here, Moniruzzaman *et al*. use metatranscriptomics to resolve interactions between giant viruses and single-celled eukaryotic hosts.

Viruses that infect marine microbes are an integral component of aquatic ecosystems, with a diversity spectrum spanning the entire Baltimore classification scheme^1^. The association of viruses with global-scale biogeochemistry, algal bloom termination events and their impact on microbial community diversity have driven scientific research in virus ecology^2^,^3^. Among these predators, giant dsDNA viruses belonging to the Nucleocytoplasmic Large DNA Virus (NCLDV) group infect single-celled eukaryotes with diverse lifestyles^4^ and are thought to be abundant in the world’s oceans^5^. Individually some of these viruses have been shown to be potential drivers of algal bloom collapse^6^,^7^. However, only a few NCLDV–host systems with established ecological relevance have been identified. As a specific example, the environmental hosts of the *Mimiviridae*, isolated using *Acanthamoeba* in the laboratory, are yet to be confirmed^8^.

Along with the NCLDVs, RNA viruses also comprise a major fraction of the marine viroplankton, infecting organisms ranging from diatoms and dinoflagellates to fish^9^. However, little is known about the ecology and host range of RNA viruses; the first RNA virus infecting a marine single-celled eukaryote was only described in 2003 (ref. 10). In addition, recent evidence suggests that a large number of novel ssDNA virus families possibly infect yet-to-be-characterized marine phytoplankton and zooplankton^11^. Collectively, these observations illustrate the strong need to develop *in situ* approaches that link the marine virosphere to their hosts within the microbial eukaryotes.

The marine ecosystem consists of complex interactions among diverse organisms and their viruses. While studying individual host-virus systems remains critical to understanding the molecular basis of interactions, studying the overall contribution of viruses in a dynamic network of organisms is hindered by methodological limitations. Culture-independent approaches to study viruses, and especially viral communities, are challenging: ‘viromes’—large metagenomic data sets enriched with viral sequences, are usually generated by size exclusion (≤0.22 μm) of bacteria and small eukaryotes^2^. This approach, however, majorly removes the large virus particles, which can range from 100 nm to ∼1.5 μm (ref. 8). Moreover, by targeting DNA, these approaches examine only the presence of particles and not their activity. Additionally, RNA-containing virus particles must be targeted separately from DNA viruses, since common methods for virus enumeration (using dsDNA intercalating stains) and DNA-based metagenomics approaches cannot detect them^12^. Nevertheless, metagenomic approaches have led to the discovery of numerous viruses, the sheer number of which greatly exceeds the viruses isolated from known hosts^13^. In contrast, metatranscriptomics has received very little attention as a tool to reveal viral activity and diversity in different ecological contexts. Among the few studies, metatranscriptomic data generated from the sub-seafloor sediments revealed gene expressions indicative of both lytic and lysogenic viral activities^14^. In a recent study, metagenomics was complemented with available metatranscriptomics data to detect the expression of viral auxiliary metabolic genes (AMGs), possibly involved in modulating host sulfur metabolism upon infection^13^.

Advances in sequencing and informatics are ongoing: while concern about chimeric assemblies and technical errors still exist, the generation of consensus genomes for populations within communities is now a common effort. Indeed, a recent workshop provided a comprehensive proposal to classify assembled virus genomes within the International Committee on Taxonomy of Viruses framework^15^. While classification of these ‘candidate’ viruses is an important step forward, connecting them to their relevant hosts will be the next major challenge. Consequently, there is a need for new toolsets to complement the current approaches and yet overcome the aforementioned issues to provide a more comprehensive picture of the viral dynamics.

Here, we examined metatranscriptomes from two highly productive sites on the east coast of USA—Quantuck Bay, New York, and Narragansett Bay, Rhode Island. Quantuck Bay experiences recurring ecosystem disruptive brown tide blooms caused by the pelagophyte *A. anophagefferens*^16^, which are shaped by a giant virus (AaV)^7^,^17^. Narragansett Bay is a highly productive system with seasonal diatom blooms, but a poorly described eukaryotic virus community. By employing selection for polyadenylation before sequencing, we were able to focus on active virus infections within eukaryotes. Using time-series data, we captured emergent relationships of putative virus–host pairings and their ecological dynamics. This approach also allowed us to characterize viruses with diverse nucleic acid genomes actively infecting eukaryotes. The results show that this approach could both confirm known virus–host infections (including the infection of *Aureococcus* by AaV) as well as identify novel virus–host interactions. These observations demonstrate that the depth of virus–host interaction in the global oceans is likely much deeper than previously anticipated, with viruses containing all forms of genetic material potentially infecting single-celled eukaryotes.

## Results

### Temporal dynamics of active giant virus infections

**Figure 1:**
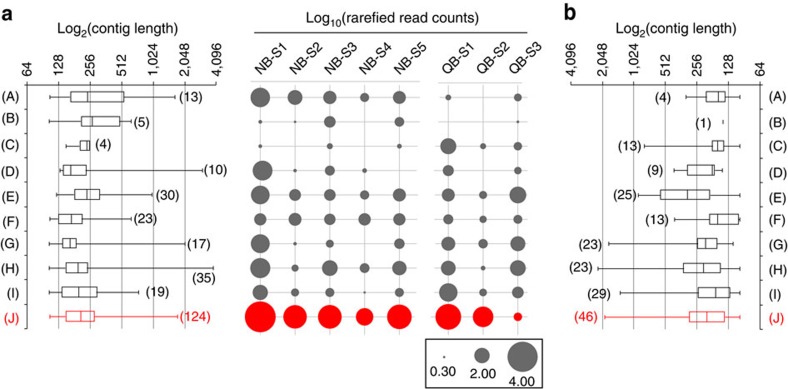
The abundance of NCLDV core genes within samples. Core genes from Quantuck Bay (**a**) and Narragansett Bay (**b**) are indicated on the *X*-axes as follows: (A) A32 virion packaging ATPase, (B) VLFT3 like transcription factor, (C) superfamily II helicase II, (D) mRNA capping enzyme, (E) D5 helicase/primase, (F) ribonucleotide reductase small subunit, (G) RNA polymerase large subunit, (H) RNA polymerase small subunit, (I) B-family DNA polymerase and (J) Major Capsid Protein (MCP). Abundance of 9 NCLDV core genes, including MCP, in terms of normalized read counts and number of contigs recovered (up to 100 bp length). The box and whisker plots represent the range of the contig lengths with number of contigs recovered for each gene in brackets. The filled circles represent the rarefied abundances of each core gene in each sample. No contigs could be detected from myristolyated envelope protein, a core NCLDV gene. MCP abundances are shown in red, while other core genes are presented in dark grey.

To identify NCLDVs, we screened contig libraries generated at each study site for 10 conserved NCLDV core genes^18^. Reads from individual samples were mapped to the core gene contigs, followed by library size normalization. At both sites, numerous contigs originating from NCLDV-specific Major Capsid Protein (MCP) were identified (Fig. 1). The abundance of reads mapped to MCP contigs was higher than the sum of mapped reads to all other NCLDV core gene contigs (Fig. 1) for all samples except QB-S3, confirming efforts suggesting that MCP is a suitable marker for NCLDV diversity^19^ and that the MCP gene is highly expressed^20^. Only distant homologues of MCP are present in *Poxviridae*^18^ and there are no homologues in recently discovered Pandora- and Pithoviruses^8^: to this end the ubiquity of this gene in all other NCLDV families makes it an excellent candidate for phylogenetic probing of metatranscriptomics data.

**Figure 2:**
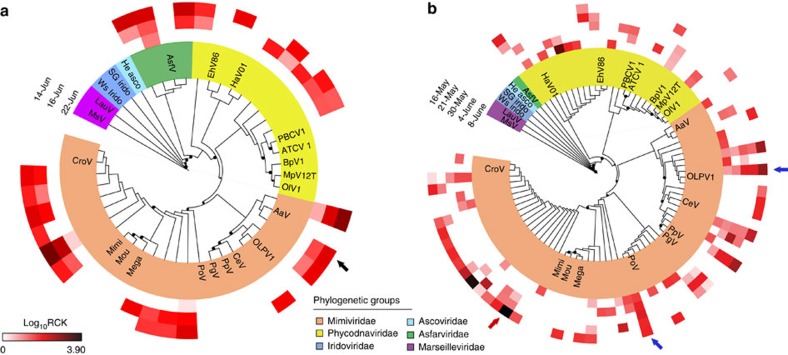
Phylogenetic reconstruction of NCLDV major capsid protein sequences. Phylogenetic placement of MCP contigs from (**a**) Quantuck Bay and (**b**) Narragansett Bay on a reference tree of NCLDVs with icosahedral capsids. Node support (aLRT-SH statistic) >50% are shown as dark circles. Contigs upto 200 bp are shown, with their expression level RCK in individual samples as a heatmap on the outer rings. Notice that the placement trees contain both AaV reference sequence and the contig originating from AaV (marked with a black arrow). The blue arrow indicates a contig with expression consistent with stable coexistence with the host/persistent infection across multiple time points, while the red arrow denotes a contig with putative ‘boom-and-bust’ like expression. Reference sequences are in bold italic typeface. Abbreviations with NCBI accession numbers: MsV-Marseillevirus (YP_003407071.1), LauV: Lausannevirus (YP_003407071.1), Ws Irido: Weisenia iridescent virus (YP_003407071.1), SG Irido: Singapore Grouper iridescent virus (YP_003407071.1), He Asco: Heliothis virescens Ascovirus (YP_003407071.1), AsfV: African swine fever Virus (NP_042775.1), EhV86: Emiliania huxleyi Virus 86 (NP_042775.1), HaV01: Heterosigma akashiwo Virus 01 (NP_042775.1), PBCV1: Paramacium bursaria Chlorella Virus 1(NP_042775.1), ATCV 1: Acanthocystis turfacea chlorella Virus 1 (NP_042775.1), BpV1: Bathycoccus prasinos Virus 1 (NP_042775.1), MpV12T: Micromonas pusilla Virus 12T (NP_042775.1), OlV1: Ostreococcus lucimarinus Virus 1 (NP_042775.1), AaV: Aureococcus anophagefferens Virus (AaV) (YP_009052173.1), CeV: Chrysochromulina ericina Virus (NP_042775.1), PpV: Phaeocystis pouchetii Virus (NP_042775.1), PgV: Phaeocystis globosa Virus (NP_042775.1), PoV: Pyramimonas orientalis Virus (NP_042775.1), Mega: Megavirus chilensis (NP_042775.1), Moumou: Moumouvirus goulette (AGF85360.1), Mimi: Mimivirus (AAV50707.1), CroV: Cafeteria roenbergensis Virus (YP_003969975.1).

We placed the MCP contigs on a reference phylogenetic tree and studied their relative expression levels in terms of a metric that we defined as ‘rarefied counts per kilobase’ (RCK) (details in Methods section). Phylogenetic placement of the contigs demonstrated that NCLDV members from *Mimiviridae* and *Phycodnaviridae* were consistently present in both Quantuck Bay and Narragansett Bay. At both sites the highest number of contigs fell within the *Mimiviridae* family, followed by *Phycodnaviridae* (Fig. 2). A large number of contigs had strong phylogenetic affinity to AaV as well as other alga-infecting members of the *Mimiviridae* clade. Their presence and relative abundance in these field surveys demonstrate that the *Mimiviridae* are an important component of the marine virosphere and are as active as the better-studied *Phycodnaviridae* group.

Brown tide bloom samples collected on 14 June (QB-S1) and 16 June (QB-S2) represented the bloom peak with an *Aureococcus* count of ∼2.28 × 10^6^ cells per ml and ∼2.23 × 10^6^ cells per ml, respectively (Supplementary Fig. 1). The third sample, collected on 22 June, represented the early stage of bloom demise, with an *Aureococcus* count of ∼1.91 × 10^6^ cells per ml. We detected a persistent infection of *A. anophagefferens* by AaV across this sampling period. High stringency (similarity ≥97%) mapping of reads to the genome identified 1,368 and 604 reads that could be assigned to peak bloom samples QB-S1 and S2, respectively, after library size normalization, while 236 reads were mapped to the QB-S3 sample taken during bloom decline (Supplementary Fig. 2). Across the entire genome, 15 AaV transcripts had more than 10 reads: roughly two-thirds of these transcripts are hypothetical, having no similarities to genes with known functions (Supplementary Data 1). Similar observations have been recorded for Mimivirus: out of 20 most highly expressed genes, 17 were found to be hypotheticals^20^. These observations suggest these genes are active during infection by AaV and other NCLDVs, and represent important targets for future studies. Notably, the AaV MCP was among the most highly expressed functional genes, with 121 total reads mapped to this gene across the three *in situ* samples from Quantuck Bay. Both total reads mapped across the AaV genome (Supplementary Fig. 1) and specifically to MCP gene (Fig. 2a) progressively declined throughout the sampling period, with the lowest number of reads mapped from S3. It may be that AaV activity was present, but predictably reduced during the bloom decline stage as host cells containing viral mRNA were destroyed—an observation further supported by a recent study where AaV amplicons were only detected during the peak of the bloom^19^. Overall, these data reinforce the utility of MCP as a marker, since the MCP dynamics was consistent with data derived from the full AaV genomic analysis (Fig. 2). Indeed, further studies are needed to be carried out in lab-based host-virus systems and in the context of diverse algal blooms, before we can link the *in situ* expression of virus genes to their impact on different stages of a bloom.

With five *in situ* samples over a period of ∼4 weeks (Supplementary Table 1), data from Narragansett Bay allowed us to observe the temporal dynamics of the NCLDVs. Some members from *Phycodna*- and *Mimiviridae* clades showed persistent evidence of infection over a prolonged period, while ‘boom-and-bust’ like relationships^4^ were possibly present for other members (Fig. 2b). For example, a number of MCP contigs were consistently expressed (within an order magnitude) between samples across time points (for example, blue arrows in Fig. 2b), an observation supporting the presence of infected hosts. While this scenario is consistent with a ‘slow-and-steady’ infection dynamic^21^, it can also be explained by persistent infections of the plankton—where ongoing virus production does not necessarily lead to host (or at least total community) mortality^22^. The expression of other phylogenetically distinct markers, however, reflected a ‘boom-and-bust’ like scenario^21^, with the expression varying across several orders of magnitude between time points. One striking example of such putative ‘boom-and-bust’ scenario was a contig in the nonalgal *Mimiviridae* family, where expression decreased by two orders of magnitudes from 16 May to 21 May and 30 May (Fig. 2b, red arrow).

### Virus infection of single-celled eukaryotes beyond NCLDVs

The marine virosphere is not limited to dsDNA viruses, as viruses containing all nucleic acid types (ss- and dsRNA as well as ssDNA) that infect marine single-celled eukaryotes have been described^9^,^11^. We extended our approach to detect the contigs that potentially originated from diverse RNA and DNA viruses other than NCLDVs. RNA viruses have a diverse size range, with Picornavirales particles as small as ∼25–30 nm^23^. Our sample collection method (Methods section) allowed detection of both ongoing virus infection (for DNA and RNA viruses) and cell-surface associated RNA viruses. It is important to mention that some (+) ssRNA viruses have poly-A tailed genomes (for example, Picorna- and Togaviruses) even outside the host^24^. Therefore, owing to their nature, the (+) ssRNA viral diversity captured by this approach reflect both actively replicating and some surface bound viruses, although this approach will largely be biased towards poly-A tail containing (+) ssRNA virus genomes, excluding those lacking poly-A tails.

**Figure 3:**
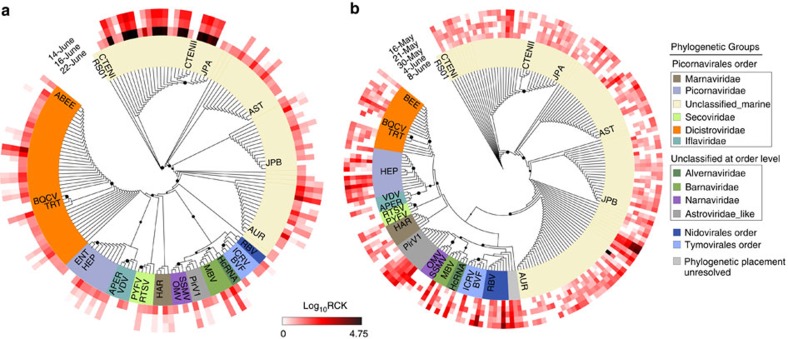
RNA-dependent RNA polymerase phylogenies for sequences attributed to (+) ssRNA viruses. Phylogenetic placement of (+)ssRNA virus contigs harbouring RNA-dependent RNA polymerase (RdRP) motifs from (**a**) Quantuck Bay and (**b**) Narragansett Bay on reference trees. Node support (aLRT-SH statistic) >50% are shown as dark circles. Contigs up to 225 bp are shown, with their expression level (rarefied read counts per kilobase—RCK) in individual samples as a heatmap on the outer rings. The reference sequences are shown in bold italic typeface. Complete name and other details of the reference sequences are presented in Supplementary Data 2.

Within our analyses, 579 and 599 contigs from Quantuck Bay and Narragansett Bay, respectively, were assigned to viruses other than NCLDVs. The majority of these contigs originated from (+) ssRNA viruses, with the main contributors coming from a yet unclassified group of viruses in the Picornavirales order^25^. Unclassified Picornavirales contigs represented 62% of the total non-NCLDV viral contigs for Quantuck Bay and 74% of this group for Narragansett Bay. Marine Picornavirales have been shown to infect diatoms (for example, *Chaetoceros* sp.*, Asterionellopsis glacialis* and *Rhizosolenia setigera)*^9^ and a marine fungoid protist, *Aurantiotrychium*^26^. The closest phylogenetic relative of this group is *Marnaviridae*, which currently have only one member—HaRNAV, that infects the marine raphidophyte *Heterosigma akashiwo*^10^. The second major group of (+) ssRNA viruses belonged to *Dicistroviridae* family, with 90 and 36 contigs from Quantuck Bay and Narragansett Bay, respectively (Fig. 3). Interestingly, the only dsRNA viruses detected in both locations were similar to viruses in the *Totiviridae, Partitiviridae* and *Hypoviridae* family, which are all known viruses of fungi^23^. These viruses may be infecting fungi that are parasitic on algae, as have been proposed recently for samples collected in the Laurentian Great Lakes^27^. While some ssDNA virus contigs from Quantuck Bay clustered with the *Nanoviridae* family, others from both locations did not form any definitive cluster with known circular DNA viruses, thus potentially representing previously undescribed circular DNA virus groups in the ocean (Supplementary Fig. 3). No (–) ssRNA viral contigs were detected^19^,^28^.

To assess how the activity of virus groups changed over time, we measured the proportion of reads that mapped to different virus groups for each library. In Narragansett Bay, the majority of the virus reads originated from the unclassified marine Picornavirales and the *Dicistroviridae*, *Secoviridae* and *Picornaviridae* families across all the time points (Supplementary Fig. 4). The unclassified marine Picornavirales group recruited from ∼68% (NB-S1) to ∼98% (NB-S5) of the non-NCLDV viral reads (Supplementary Fig. 4). In Quantuck Bay, reads from both unclassified marine Picornavirales and ssDNA viruses dominated libraries during the first two time points (Supplementary Fig. 5). However, a shift in the proportional abundance of virus reads was observed on the third day (QB-S3), when the unclassified marine Picornavirales became dominant (93% of the non-NCLDV virus transcripts, Supplementary Fig. 5). Overall, 2.4% of the entire QB-S3 library (∼4.3 million fragments) mapped to these unclassified Picornavirales, relative to 0.043 and 0.027% of reads for QB-S1 and QB-S2, respectively. This indicated a striking increase, concordant with the decline of the brown tide bloom. Phylogenetic analysis confirmed these ssRNA viral contigs to be consistent with the unclassified Picornavirales group (Fig. 3a). *Aureococcus* blooms include diatoms, dinoflagellates and high densities (∼10^4^ cells per ml) of heterotrophic protists alongside *Aureococcus*^19^,^29^. The striking increase in the unclassified Picornavirales could be related to infection of a host that co-occurs with *Aureococcus* and the potential shift in competition that might occur during *Aureococcus* bloom decline. These observations suggest a broad ecological role for viral infection during phytoplankton bloom decline, which would not have been resolved with targeted studies of AaV or metagenomic approaches. Taken together, the apparent dynamics and abundance of this unclassified Picornavirales suggest this group is a major component of the marine virioplankton, and strengthens previous observations that the Picornavirales phylogenetically distinct from the established families can be dominant members in different marine environments^12^,^30^. Owing to their small size, detection and quantification of RNA viruses and ssDNA viruses pose significant technical challenges^30^. Our results, however, clearly point to the power of screening metatranscriptomes for the simultaneous analysis of the dynamics of a large cross-section DNA and RNA viruses.

**Figure 4:**
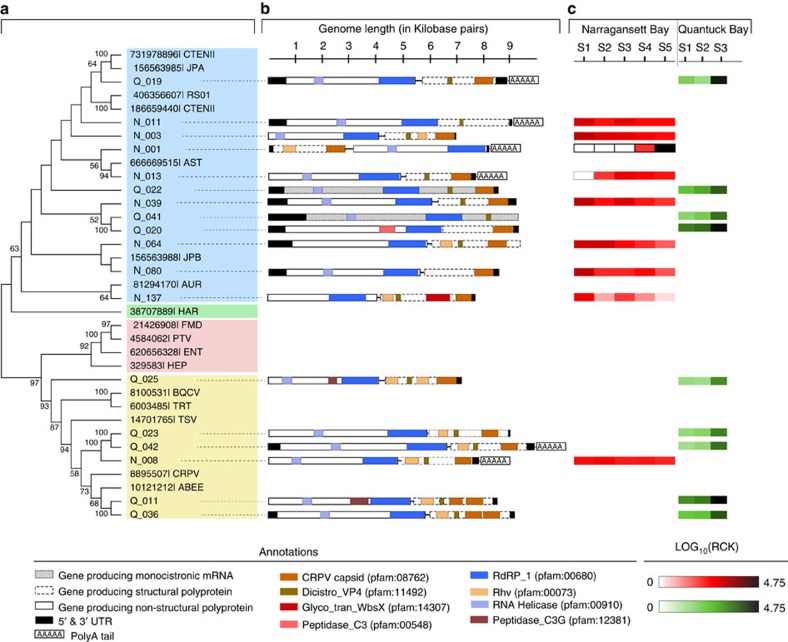
Complete or near-complete Picornavirales genomes recovered from Quantuck Bay and Narragansett Bay study sites. Panel (**a**) shows the phylogenetic classification of these contigs in a topology-only maximum likelihood tree, with contigs from Quantuck Bay having prefix ‘Q_’ and contigs from Narragansett Bay having prefix ‘N_’. Panel (**b**) shows the genome architecture of these contigs with protein domains and putative CDSs. Panel (**c**) shows the expression level of these (rarefied read counts per kilobase—RCK) viruses in across different *in situ* samples.

Eighteen of the assembled contigs (nine from each site) were >7,000 bp and had best hits to different Picornavirales members. Phylogenetic analysis based on the RNA-dependent RNA polymerase (RdRp) gene and feature analysis of existing (+) ssRNA virus genomes suggested that these contigs are complete or near-complete Picornavirales genomes (Fig. 4). While the possibility of errors in assembly (for example, chimeras) from a mixed community cannot be ignored, bioinformatic analyses support the legitimacy of these genomes. The domain architecture of the protein coding genes in these genomes match the known (+) ssRNA virus domain organizations. Specifically, the domains characteristic of structural and nonstructural CDSs are restricted to the corresponding CDSs (Fig. 4). It is likely, however, that on a base-by-base level any individual viral genomes assembled from environmental data sets (whether metagenomes or metatranscriptomes) could represent ‘genomic averages’ (or a genomic composite) of multiple strains of the same virus, essentially ignoring the strain-specific heterogeneities (that is, single nucleotide polymorphisms). Sixteen of these genomes were dicistronic—they harboured two ORFs coding for structural and nonstructural proteins, while the remaining two were monocistronic (Fig. 4), revealing differences in genome architecture among group members (Fig. 4). Remarkably, one virus (N_001) had a reverse orientation of the genes with the first ORF encoding for the structural protein, which is unusual for dicistronic Picornavirales^31^. In addition, a glycosyl transferase domain was found in N_137 (Fig. 4). To our knowledge, the presence of glycosyltransferase domains has only been reported in members of the *Endornaviridae* family dsRNA viruses^32^.

We also tracked the dynamics of these *de novo* assembled genomes by mapping the data collected over spatiotemporal gradients. All the (+) ssRNA virus genomes from Quantuck Bay samples showed higher relative expression during bloom decline (QB-S3) compared to the time points corresponding to the bloom peak (Fig. 4, panel C). N_001, a candidate virus from Narragansett Bay, was not present in the first three time points. Its expression was only observed during the fourth sampling point, which was followed by a dramatic increase during the last sampling point, when it recruited ∼0.55% of the reads from the entire library (Fig. 4, panel C). The closest known phylogenetic relative of N_ 001 is a virus infecting diatom *A. glacialis* (Fig. 4), suggesting the putative host may be a diatom. Narragansett Bay was experiencing a spring diatom bloom during the sampling period with ‘boom-and-bust’ abundance cycles in the relative abundance of putative diatom hosts^28^, consistent with these observed viral dynamics.

## Discussion

This study presented the opportunity to evaluate relationships among diverse single-celled eukaryotes and their pathogens, with the established *AaV-Aureococcus* association acting as a *de facto* internal standard. Transcripts from DNA viruses must originate within the host cells, and thereby, for a particular host-virus pair, a significant and strong positive correlation is to be expected for gene expression. Building on this idea, host gene expression of at least a subset of the host’s genome is a prerequisite to observe gene expression of a virus specific to that host, as evidenced by transcriptomic landscape of host-virus dynamics in culture^20^,^33^ and induced blooms in mesocosms^34^. To expand our data, we also took advantage of concurrent nutrient amendment studies in mesocosms (see Methods section), which provided additional samples for our analyses. Brown tides are characterized by high abundance of other single-celled eukaryotes alongside *Aureococcus*^19^. At the same time, a dynamic community harbouring diatoms, dinoflagellates, chlorophytes and ochrophytes was observed in the Narragansett Bay samples analysed in this study^28^. Thus, the protist communities in these sites potentially include the hosts of the viruses that we detected.

**Figure 5:**
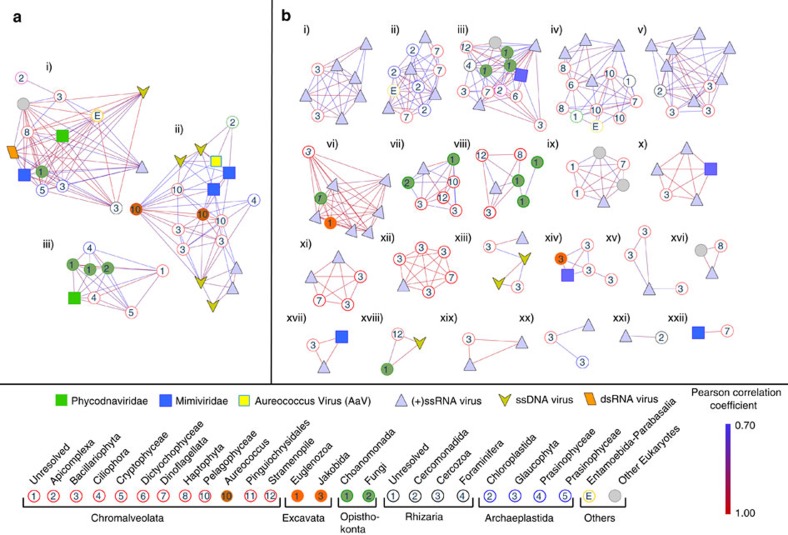
SIMPROF clustering of virus and eukaryotic markers from Quantuck Bay and Narragansett Bay study sites. Statistically significant SIMPROF clusters containing both viral and eukaryotic members from (**a**) Quantuck Bay and (**b**) Narragansett Bay. Contigs are shown as nodes and the Pearson’s correlations as the connecting edges. Within the SIMPROF clusters, correlations only significant at the level of *P*≤0.1 are shown. Phylogenetic classifications of the contigs are shown in the bottom panel. *A. anophagefferens* (dark brown circles) and A. anophagefferens Virus (bright yellow square) are in cluster A(ii).

We inspected statistical co-occurrences among the contigs containing virus and eukaryote-specific marker genes based on their expression patterns. Since the poly-A-selected metatranscriptomes are largely depleted of ribosomal RNA marker genes, we employed functional genes suitable for phylogenetic analysis. Expression of MCP (dsDNA NCLDVs), RdRP (RNA viruses) and viral replicase (ssDNA viruses) were compared to the functional eukaryotic marker gene RNA polymerase II large subunit (RPB1, Supplementary Fig. 6), a candidate gene to resolve the phylogenetic history of different eukaryotic lineages^35^,^36^. Hierarchical clustering of a Pearson’s correlation matrix followed by SIMPROF analysis^37^ was used to detect statistically distinct clusters that contained both virus and eukaryotic marker genes that could be classified into phylogenetic groups. This analysis produced several statistically distinct clusters harbouring both viral and eukaryotic contigs (Fig. 5). A single cluster (Fig. 5a(ii)) harboured both AaV and *Aureococcus*, validating that established ecologically relevant relationships between viruses and their hosts can be retrieved using this approach.

Close inspection revealed other interesting relationships among the coexisting eukaryotic and viral components. Cluster A(ii), while containing both *Aureococcus* and AaV, also contained another *Mimiviridiae* member, several ssDNA and (+) ssRNA viral contigs along with eukaryotes belonging to prasinophyceae and pelagophyceae (Fig. 5). The possibility of *Aureococcus* being infected by more than one virus type cannot be discounted (and indeed is perhaps likely). Moreover, the potential for AaV to infect closely related pelagophytes remains a possibility (although this has not been seen in lab studies)^38^. One cluster, A(i), which contained both a *Phycodna*- and a *Mimiviridae* member, also included a RPB1 contig phylogenetically placed in the Cercozoa group (Fig. 5). Although no cercozoan host-NCLDV pairs currently exist in culture, a recent study showed integration of NCLDV genes in the genome of a cercozoan *Bigelowella natans*^39^. This integrated NCLDV in the *B. natans* genome potentially belongs to the *Phycodnaviridae*, as revealed by the same study through phylogenetic analysis of the MCP gene. Our observation supports the possibility that there might be yet to be discovered cercozoan-Phycodnaviridae interactions.

Similar clusters of phylogenetically distinct eukaryotes and viruses were also found in Narragansett Bay. For example, cluster B(iii) contained a *Mimiviridae* and several ssRNA viral contigs connected to choanomonada, stramenopile, diatom and dinoflagellate members (Fig. 5). The majority of the eukaryotic contigs belonged to diatoms and dinoflagellates in the Narragansett Bay samples, which reflects the composition of single-celled eukaryotes in this site^28^. A large number of contigs having phylogenetic affinity to choanomonada were found in both Quantuck Bay and Narragansett Bay locations and were represented in several of the representative SIMPROF clusters (Fig. 5). While larger networks of viruses and eukaryotes also existed, clusters with fewer members revealed more specific relationships. For example, cluster B(xiv) contained one *Mimiviridae*, one jakobida (heterotrophic flagellate) and several diatom contigs (Fig. 5). To date the obligate heterotrophs known to be infected by *Mimiviridae* members are *Cafeteria roenbergensis*^40^, *Acanthamoeba*^41^ and *Vermaamoeba* spp^42^. Additionally; Cluster B(xviii) harboured a ssDNA virus, a stramenopile and a choanomonada member, while cluster B(xxii) revealed a one-to-one relationship between a *Mimiviridae* and a dinoflagellate (Fig. 5). Only one dinoflagellate—*Heterocapsa circularisquama*—has been shown to be infected by a NCLDV^43^, so this potential host-virus pair is of particular note. Cluster B(x) and B(xvii) consisted of *Mimiviridae*, diatoms and ssRNA viruses. No diatom is yet known to be infected by a NCLDV, although a large number of ssRNA viral contigs in our study are phylogenetically close to diatom-infecting RNA viruses in the unclassified marine picornavirales group (Fig. 3). A number of clusters (for example, B(xii)) were enriched with both ssRNA virus and diatom contigs. These relationships between ssRNA viruses and the eukaryotes need to be interpreted with caution, however, as these contigs might originate both from free virus particles and/or viruses within hosts.

Several clusters also contained fungal contigs along with other eukaryotes—pointing to the possibility of broad parasitic relationships with phytoplankton and other single-celled eukaryotes. The AaV-*Aureococcus* cluster A(ii) harboured a fungal contig and a *Barnaviridae* member—a virus family with fungi as the only known hosts (Fig. 5)^23^. Several other clusters, for example, A(iii) and B(vii) also contained fungal contigs. While such observations are not definitive, they point to the existence of parasitic relationships resulting in complicated ecological interactions involving unicellular eukaryotes, fungi and fungal viruses in marine ecosystems^27^.

Increased sample resolution in the future will resolve more statistically robust relationships, which can further narrow potential interacting partners. One limitation of reference-independent assembly of high-throughput data is fragmented contigs originating from same transcript—which is illustrated by two *Aureococcus* specific RPB1 contigs in cluster A(ii) that originated from a single coding sequence. Increased sequencing, along with the continued development of assembly tools, will provide better resolution to these relationships. These limitations notwithstanding, the analysis provides a ‘proof-of-principle’ for inferring the complex relationships among diverse unicellular eukaryotes and their viruses using metatranscriptome data.

In this study, we demonstrate how metatranscriptomics can provide a unique view of the marine virosphere by simultaneously detecting multiple viral infections across the landscape of the eukaryotic plankton within an ecosystem setting. This effort can largely overcome previous technical limitations involved in the study of different viral groups, owing to their size range and genome type, within the same sample. In the last two decades, we have learned much regarding the diversity and dynamics of the phages in the ocean, but the eukaryotic virosphere has remained elusive, with little known about who is infecting whom in the environment^44^. As demonstrated in our study, analysing the vast wealth of information captured by metatranscriptomics, in a statistical framework, can be an important step towards answering this vital question.

## Methods

### Experimental design

Samples were collected from a brown tide bloom in Quantuck Bay (Latitude=40.806395; Longitude=−72.621002), NY that occurred from late May to early July, 2011, covering the initiation, peak and demise of the bloom. Samples collected on June 14 (BT-S1) and June 16 (BT-S2) represented the peak of the bloom, while sample collected on June 22 (BT-S3) represented the initial phase of bloom decline. *Aureococcus* cells were counted from glutaraldehyde (1% final v/v) fixed whole water samples using an enzyme-linked immunosorbent assay (ELISA) with a monoclonal antibody as described previously^45^. Briefly, 1:1,000 fold dilution of the environmental samples were made in phosphate buffer saline Tween 20. The samples were stained with the fluorescein isothiocyanate conjugated monoclonal antibody for 30 min. Aureococcus cells were then identified and counted if they fell within a defined region in the side scatter—FL1 (green light, 500–560 nm) plot. *In situ* samples from June 22nd (third sampling point) was also used to carry out nutrient amendment experiments—results from these samples were included to both expand the environmental conditions sampled (for a similar population) as well as to increase the total number of individual samples (and thus statistical power) for analyses. Briefly, bottles were filled with natural sea water from the bloom and were amended with 25 μM ammonium only (+N), 4 μM phosphate only (+P) and 25 μM ammonium and 4 μM phosphate (+N&P) in triplicate. Three additional bottles with no nutrient addition were used as control. The samples were then incubated for 24 h in a floating chamber at 0.5 m in eastern Shinnecock Bay at the Stony Brook—Southampton Marine Science Center under one layer of neutral density cover to mimic the light and temperature levels of Quantuck Bay. Samples for *Aureococcus* cell density measurement and total RNA extraction were collected at *T*=0 and *T*=24 h. Approximately 25 ml of natural seawater from each of the *in situ* and nutrient amendment samples were prefiltered through 5 μm polycarbonate (PC) filters and collected on 0.2 μm PC filters. The samples were flash frozen immediately after filtration and transferred to −80 °C. Prior RNA extraction, CTAB buffer (Teknova, CA, USA) amended by polyvinylpyrrolidone (1% mass/vol) was added to each of the samples.

The detailed sampling procedure for Narraganset Bay has been previously described in Alexander *et al*.^28^. Samples were collected from a long term sampling site in Narragansett Bay (41°34′12′′ N, 71°23′24′′ W) during 2012 on May 16 (NB-S1), May 21 (NB-S2), May 30 (NB-S3), June 4 (NB-S4) and June 8 (NB-S5). Sample collection and processing was completed within 0830 and 0900 local time to reduce the influence of diel signals. Two litre of water from each sample was filtered on 5.0-μm pore size PC filters using a peristaltic pump. The filters were snap frozen in liquid nitrogen and stored at −80 °C until RNA extraction. Water collected along with NB-S3 was also used for nutrient amendment experiments. For this, triplicate 2.5 l bottles were filled with water prefiltered through a 200-μm mesh and amended with specific nutrients to create +N, +P, –N, –P treatments alongside an ambient control. The +N and +P treatments were designed to eliminate nitrogen and phosphate stress signals, whereas the –N and –P treatments were designed to drive the treatments towards each limitation, respectively, by skewing the nutrient ratios^28^. N and P amendment concentrations were ∼10-fold the seasonal average N and P concentrations measured at the station II in the surface waters of Narragansett Bay. The +P and +N amendment contained 3 μM phosphate and 10 μM nitrate, respectively. The –P amendment contained 10 μM nitrate, 68 μM silica, 4.6 μM iron and f/5 vitamins. The –N treatment was amended with 3 μM phosphate, 68 μM silica, 4.6 μM iron and f/5 vitamins. The f/5 media ratios^46^ were followed for silica, iron and vitamin amendments. Bottles were incubated for 48 h in a flow-through incubator at ambient temperature and photosynthetically active radiation. After the end of the incubation, treated and control samples were filtered and stored for RNA extraction in the same manner for the *in situ* samples.

### RNA extraction and sequencing

RNA was extracted from Quantuck Bay samples using the UltraClean Plant RNA Isolation Kit (MO BIO Laboratories, CA, USA) according to manufacturer’s protocol. RNA samples were quantified spectrophotometrically and were sequenced in the Columbia Sequencing Center (NY, USA) using Illumina HiSeq platform with poly-A enrichment at a depth of ∼50 million 100 bp single end reads. Two more replicate samples were sequenced from June 22 (QB-S3) at a depth of 100 million reads. For the present study, these biological replicates from QB-S3 were pooled together before further analysis.

For Narragansett Bay, replicate filters from each treatment and *in situ* samples were pooled, representing 6 l of water for each sample. RNA was extracted using RNeasy Mini Kit (Qiagen, Hilden, Germany) according to a modified yeast RNA extraction protocol. Briefly, lysis buffer and RNA-clean zircon beads were added to the filter. Samples were then vortexed for 1 min, placed on ice for 30 s, and then vortexed again for 1 min. The resulting RNA was eluted in water and possible DNA contamination was removed using a TURBO DNA-free Kit (Thermo Fisher Scientific, MA, USA). RNA from each triplicate was pooled by sample or treatment. >1,000 ng RNA from each sample then went through a poly-A selection using oligo-dT beads followed by library preparation with TruSeq RNA Prep Kit (Illumina, CA, USA). The samples were sequenced with an Illumina HiSeq2000 at the Columbia University Genome Center to produce ∼60 million; 100 bp paired-end reads per sample.

### Read assembly and screening

For bioinformatics analyses, sequence reads from both locations were quality trimmed (stringent trimming (quality score ≤0.03), No ‘N’s allowed, 70 bp size cutoff) in CLC genomics workbench 8.0 (Qiagen, Hilden, Germany). The data from all the Quantuck Bay samples were combined and assembled together, and a similar assembly procedure was also followed for the Narragansett Bay specific samples. This resulted in 2,455,926 contigs for Quantuck Bay and 9,525,233 contigs for Narragansett Bay at a 100 bp size cutoff.

Most giant virus genomes contain multiple genes having domains characteristic of viral capsids. Among the capsid orthologues, protein ‘VP54’ (in *Paramacium bursaria Chlorella* Virus—PBCV, NCBI accession: BAA22198) and ‘D13L’ protein of Mimivirus (NCBI ID: AAV50707) have been experimentally and bioinformatically characterized to be the MCP^47^,^48^, which encode the monomer of the icosahedral capsid of these viruses. One orthologue in other Phycodnaviridae and Mimiviridae members shows high similarity to PBCV VP54 and Mimivirus D13L, implying a common ancestor^49^,^50^. As well, the genomes of *Marseilleviridae*, *Iridiovirida*e and *Ascoviridae* members have only a single capsid homologue annotated as MCP. Finally, representative Asfarviridae member African swine fever virus harbours a single capsid gene, also known to encode the major structural component^51^. On the basis of this information, we first screened for putative MCP orthologues (one in each virus) in the sequenced NCLDVs from different families. A HMM profile was created after aligning the amino-acid sequences of the detected MCP reference orthologues. This HMM profile was queried against the translated contig libraries from Quantuck Bay and Narragansett Bay to select the putative MCP candidate contigs using HMMER^52^. Contigs identified went through an additional screening step—they were searched using BLASTP against the NCBI nr database. Those with best hits only to the putative MCP orthologues were retained for analysis.

For selecting eukaryotic RPB1 contigs, HMM profile specific to domain ‘RPB1-C-term (NCBI CDD ID: cd02584)’ and ‘RPB1-N-term (NCBI CDD ID: cd02733)’ was used to query the contig libraries. All the MCP and RPB1 candidate contigs detected in this manner were queried against NCBI Refseq database and only contigs with first BLASTx hits (e-value cutoff ≤10^–3^) to MCP and RPB1 were kept for further analysis.

To detect contigs originating from viruses other than NCLDVs, we combined the proteins derived from all the viruses having algal, fungal and protozan hosts available on NCBI database. This protein database was queried against the contig libraries using tBLASTn with an e-value cutoff of ≤10^–3^. All the candidate contigs screened by this procedure were then queried against NCBI Refseq database using BLASTx. Only contigs having topmost hits to different viruses were kept for further analysis. All these contigs had best hits to diverse eukaryotic viruses- no hits to prokaryotic viruses were detected. This is probably due to the nature of the query data set, which contained proteins exclusively from viruses infecting algae, fungi and protists. These virus contigs were binned into distinct viral groups according to their best BLASTx hits. Percentage of reads recruited to individual viral groups was calculated for determining proportional abundance of different viral groups over different time points. For detailed phylogenetic analysis of ssRNA and ssDNA viruses, subset of these contigs harbouring RdRP (pfam id: PF05183) and viral replicase (pfam: PF03090) motif were selected using HMM profile specific to these motifs.

### Genomic and phylogenetic analysis

To reconstruct phylogenies, reference sequences for MCP (giant viruses), RdRP (RNA viruses), viral replicase (ssDNA virus) and RPB1 (eukaryotes) were downloaded from NCBI Refseq database. A number of RPB1 sequences representing several eukaryotic groups were also collected from Marine Microbial Eukaryotes Transcriptome Sequencing Project (MMETSP)^53^ peptide collections, for which no representative genomes are available. The reference sequences were aligned in MEGA 6.0 (ref. 54). Maximum likelihood phylogenetic trees were constructed in PhyML^55^ with LG model, gamma shape parameter and frequency type estimated from the data. aLRT-SH like statistic was calculated for branch support. The eukaryotic classification scheme by Adl *et al*.^56^ was followed. Selected contigs were translated to amino-acid sequences and were placed on the reference trees in a maximum likelihood framework using pplacer^57^. The placement files were converted to trees with pendant edges showing the best placement of the contigs using ‘guppy’ tool of pplacer. The placement trees were visualized and annotated using iTOL interface^58^.

For the complete or near-complete Picornavirales genomes ORFs were predicted on using CLC genomic workbench 8.0 (www.clcbio.com). The genome annotation with predicted features was assisted by pfam^59^ and Conserved Domain Database (CDD)^60^ search.

### Statistical analysis

Quality trimmed reads were mapped to the selected viral and eukaryotic contigs from individual read libraries with high stringency (97% identify, 70% length fraction matching) in CLC genomics workbench 8.0. The read mapping values were normalized by library size and length and expressed as RCK. RCK values of viral and eukaryotic contigs >225 base pairs were converted into matrices separately for Quantuck Bay and Narragansett Bay data sets, which included mapping statistics from both *in situ* and nutrient amendment libraries. Group averaged hierarchical clustering was performed on these matrices using Pearson’s correlation coefficient in PRIMER 7.0. SIMPROF test^37^ was applied on the clusters with 5% significance level and 1,000 permutations to identify statistically distinct clusters. Selected clusters were visualized and annotated in Cytoscape 3.0 (ref. 61).

### Data availability

The field sequence data reported in this paper have been deposited in the National Center for Biotechnology Information Sequence Read Archive, www.ncbi.nlm.nih.gov/sra (Narraganset Bay accession no. SRP055134; Quantuck Bay accession no. SRP072764). The genomes are submitted in the NCBI database under the accession numbers: KY286099—KY286107 and KY130489—KY130497.

## Additional information

**How to cite this article:** Moniruzzaman, M. *et al*. Virus-host relationships of marine single-celled eukaryotes resolved from metatranscriptomics. *Nat. Commun.* **8,** 16054 doi: 10.1038/ncomms16054 (2017).

**Publisher’s note:** Springer Nature remains neutral with regard to jurisdictional claims in published maps and institutional affiliations.

## Supplementary Material

Supplementary Information

Supplementary Dataset 1

Supplementary Dataset 2
