# Supplementary material for: Virus-host relationships of marine single-celled eukaryotes resolved from metatranscriptomics
Source: Nat Commun. 2017 Jun 28;8:16054. doi: 10.1038/ncomms16054 (PMC5493757; doi:10.1038/ncomms16054)
Supplement: Supplementary Information [file ncomms16054-s1.pdf]

Title of file for HTML: Supplementary Information
Description: Supplementary Figures and Supplementary Table

Title of file for HTML: Supplementary Dataset 1
Description: Mapping statistics of reads from the Quantuck Bay libraries to the genes of Aureococcus anophagefferens virus (AaV).

Title of file for HTML: Supplementary Dataset 2
Description: Abbreviations and NCBI accession numbers of the reference sequences used to reconstruct the Eukaryotic RPB1, viral RdRP (+ssRNA virus) and viral replicase (ssDNA virus) phylogenies.

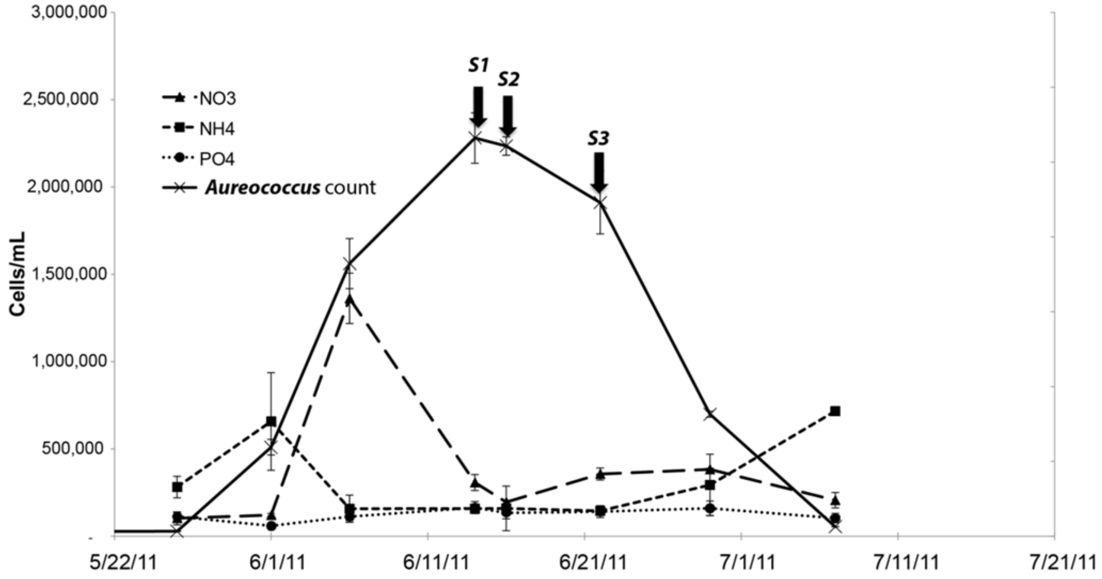

Supplementary figure 1: – *Aureococcus* and nutrient dynamics during different phases of the brown tide bloom. The samples collected for metatranscriptomic analysis are marked with black arrows.

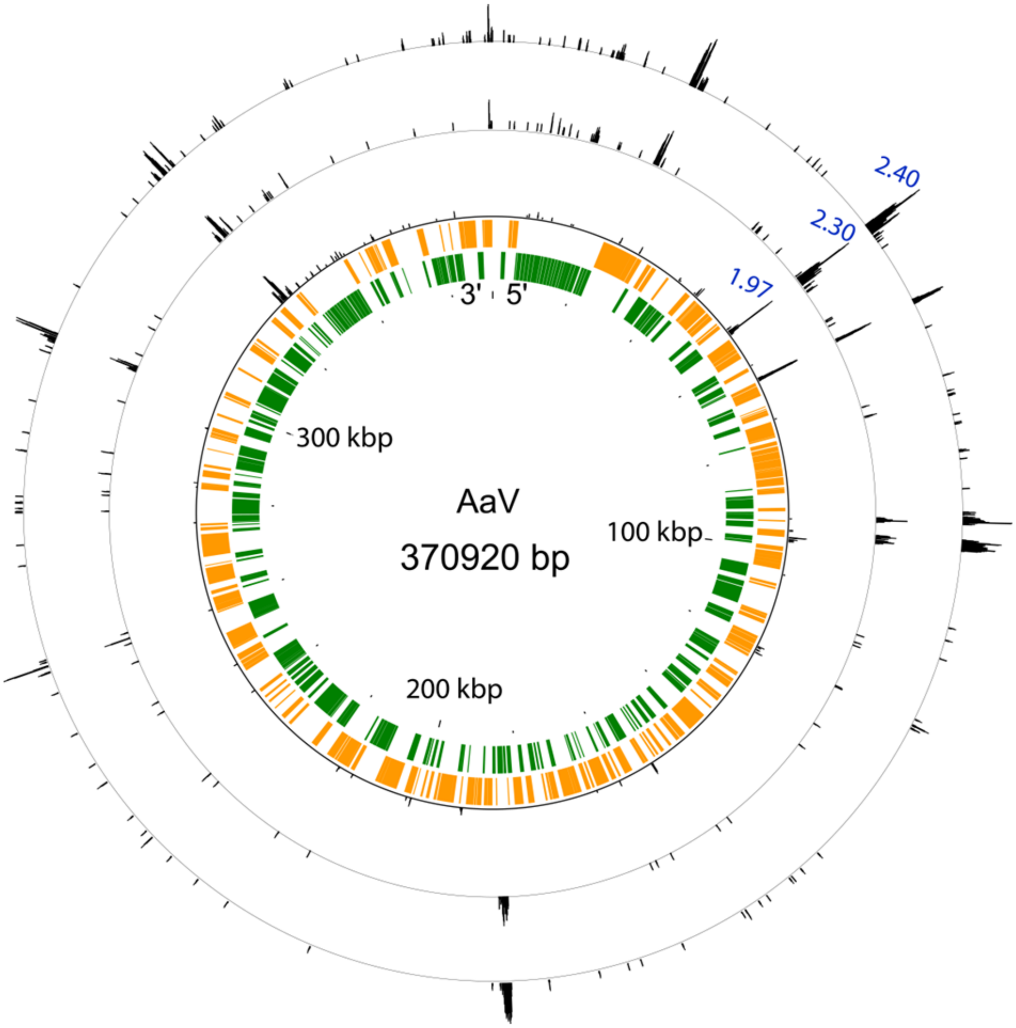

Supplementary Figure 2: Normalized read mapping statistics to the 370,920 bp AaV genome from the three Quantuck Bay samples. From outermost to innermost ring: Log converted number of reads mapped from QB-S1 (June 14), QB-S2 (June 16) and QB-S3 (June 22), respectively. Forward CDSs are in green while reverse CDSs are shown in orange. The value in blue on each ring represents the highest number of reads (log converted) mapped from the particular sample.

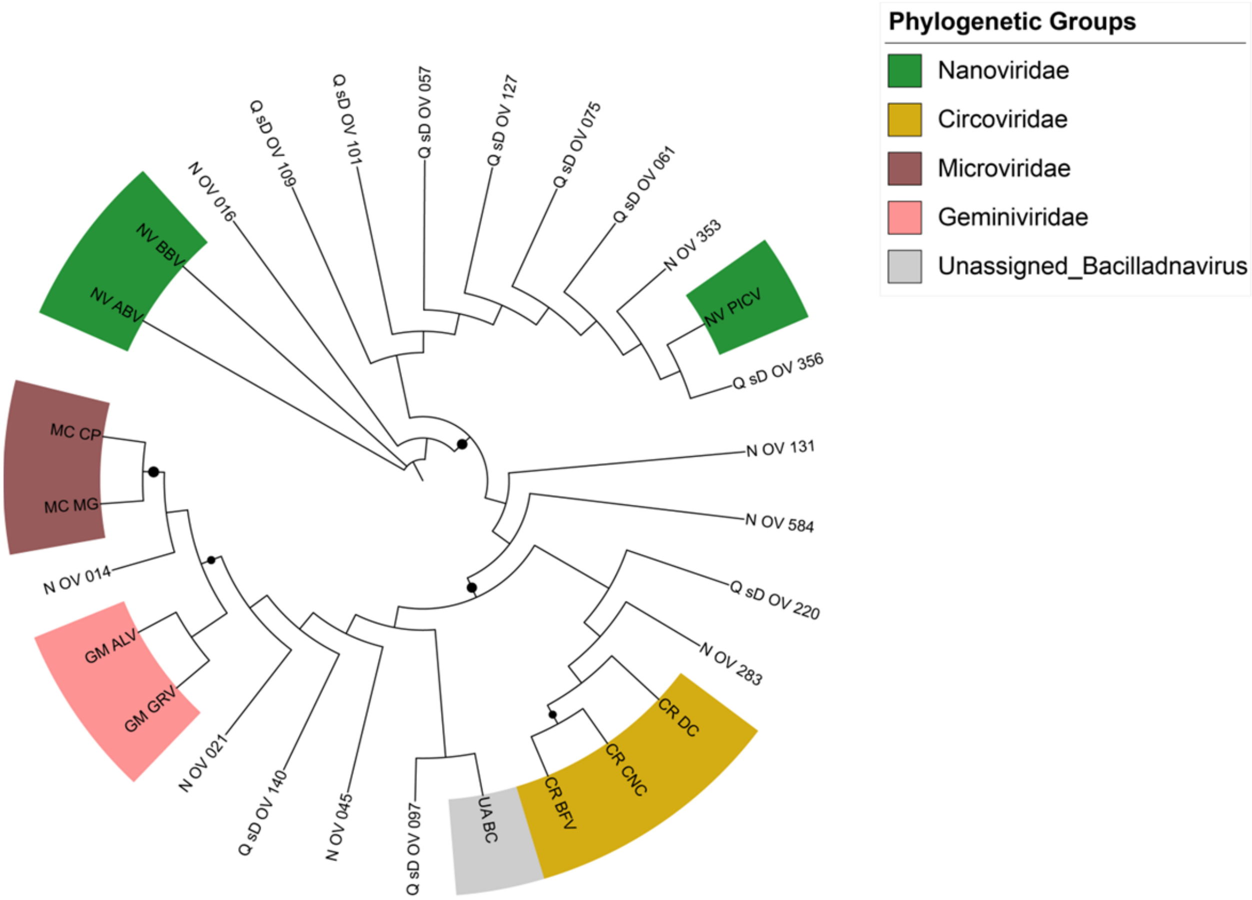

Supplementary Figure 3: Phylogenetic placement of ssDNA virus contigs (prefix: N_OV_ and Q_sR_OV_) harboring viral replicase motifs from Quantuck Bay and Narragansett Bay on a reference tree. Node support (aLRT-SH statistic) >50% are shown as dark circles. Contigs up to 225bp are shown. The reference sequences are shown in bold italic typeface. Complete name and other details of the reference sequences are presented in Supplementary Data 2.

Marnaviridae	0.707	0.353	0.325	2.225	0.028
Other Picornavirales	0.234	0.099	0.171	0.304	0.004
Narnaviridae	0.904	0.105	0.330	0.969	0.045
Alphatetaviridae	0.881	0.277	0.495	2.521	0.090
Virgaviridae	0.032	0.127	0.413	1.971	0.146
Potyviridae	0.000	0.006	1.992	0.000	0.000
PirV-1 like	3.948	1.490	1.821	1.076	0.511
Fusariviridae	0.000	0.000	0.000	0.000	0.001
Alvarnaviridae	0.042	0.006	0.001	0.008	0.000
Totiviridae	0.017	0.108	0.003	0.074	0.008
Partitiviridae	0.002	0.095	1.016	0.074	0.017
ssDNA virus	2.350	3.963	1.484	5.206	0.300
DNA-RNA hybrid virus like	0.000	0.000	0.002	0.000	0.002
Bacillaridnavirus	0.065	0.115	0.431	0.895	0.048

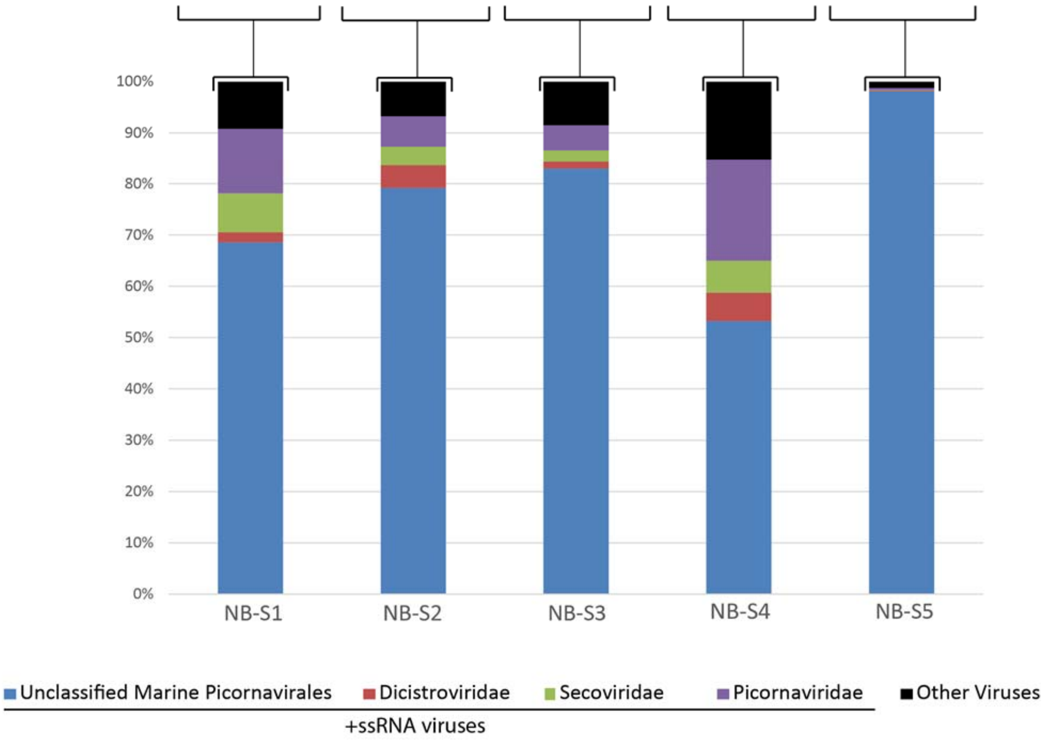

Supplementary Figure 4: Proportional abundance of reads (in percentage) assigned to different viral groups in the Narragansett Bay study site over the five *in situ* samples. The 'other viruses' portion (in black) is further broken down into viral groups in the tables, which represent a minor contribution towards the diversity.

	QB-S1	QB-S2	QB-S3
Marnaviridae	0.075	0.176	0.025
Secoviridae	0.155	0.203	0.072
Picornaviridae	0.107	0.065	0.012
Other Picornavirales	0.767	1.007	2.824
Potyviridae	0.895	1.091	0.057
PirV-1 like	0.330	0.545	0.030
Fusariviridae	0.000	0.000	0.005
Alvarnaviridae	0.005	0.000	0.004
Tymovirales	0.000	0.000	0.000
Nodaviridae	0.000	0.000	0.000
Fisavirus like	0.032	0.083	0.016
Halastavi arva RNA virus like	0.037	0.203	0.042
Totiviridae	0.000	0.000	0.001
Partitiviridae	0.021	0.028	0.000
Hypoviridae	0.037	0.120	0.003
Unclassified Mycovirus	0.053	0.037	0.016

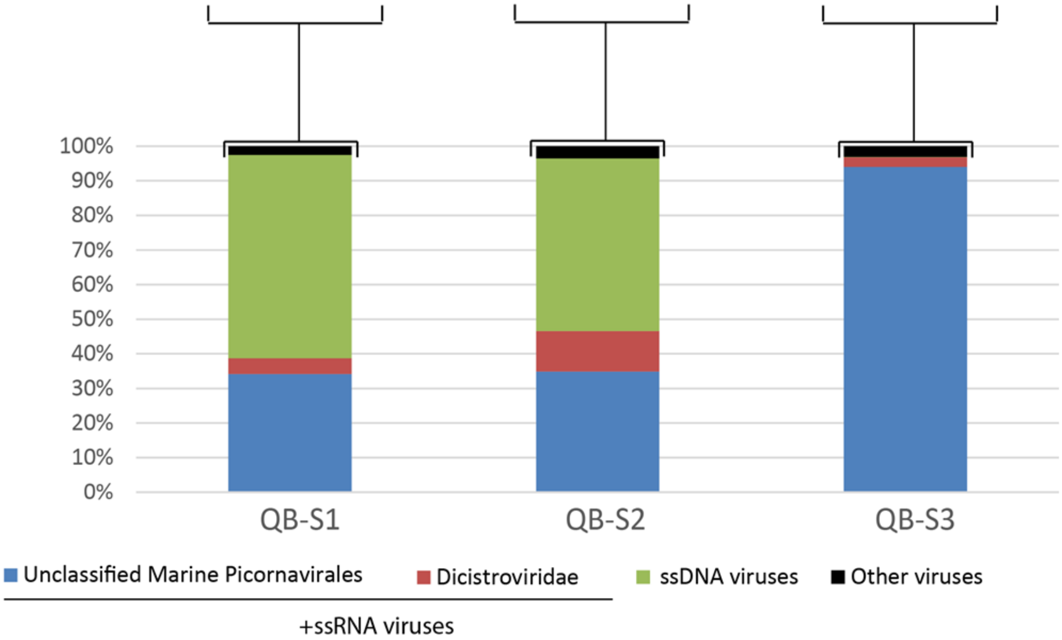

Supplementary Figure 5: Proportional abundance of reads (in percentage) assigned to different viral groups in the Quantuck Bay study site over the three *in situ* samples. The ‘other viruses’ portion (in black) is further broken down into viral groups in the tables, which represent a minor contribution towards the diversity.

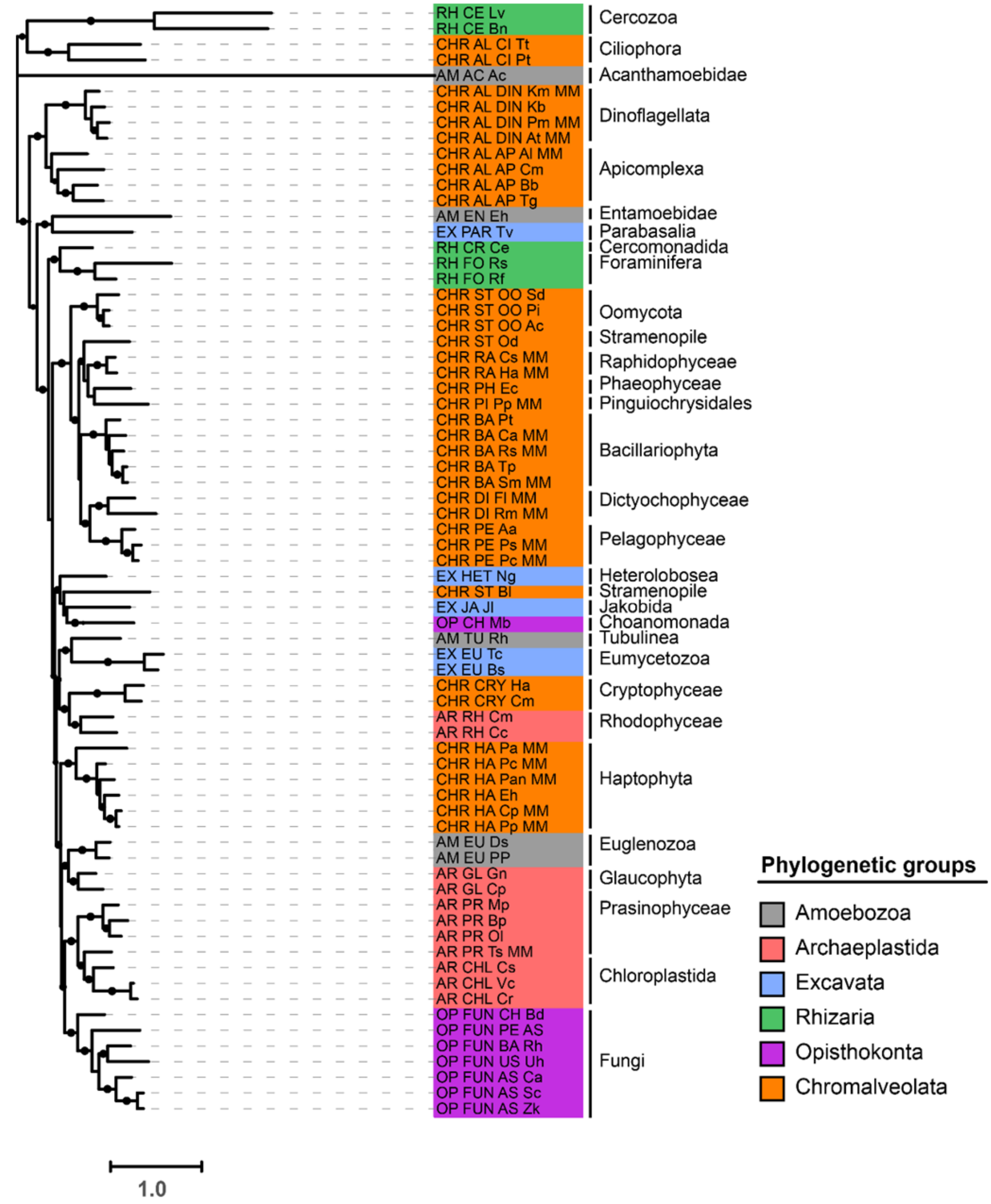

Supplementary Figure 6: Maximum likelihood phylogenetic tree of eukaryotic RPB1 (RNA polymerase II large subunit) used as reference to classify the RPB1 contigs assisted by pplacer. Node support (>50%) values >50% are shown as dark circles. Complete names and other details of the reference sequences are provided in Supplementary Data 2.

Supplementary Table 1: RNA–seq library sizes after quality trimming. The Quantuck Bay samples were sequenced as single reads, while paired-end sequencing was carried out for the Narragansett Bay samples (denoted by X2). For Quantuck bay, the nutrient amendments were carried out in bottles filled with natural seawater from the bloom and amended with the following nutrients in triplicate: 25 µM ammonium only (+Nitrogen), 4 µM phosphate only (+Phosphorus), and 25 µM ammonium and 4 µM phosphate (+Nitrogen and +Phosphorus). Three additional bottles were filled and no nutrients were added (control). Similar amendments were conducted for the Narragansett Bay samples (see Alexander *et al*., 2015).

	Sample	Number of reads
Quantuck Bay	June 14, 2011 (QB-S1)	43,755,618
	June 16, 2011 (QB-S2)	40,228,517
	June 22, 2011 (QB-S3)	214,681,748
	Nutrient amendment -control	41,156,535
	+Nitrogen	39,539,666
	+Phosphorus	36,797,290
	+Nitrogen and +Phosphorus	37,442,936
Narragansett Bay	16-05-2012 (NB-S1)	88,207,422 X2
	21-05-2012 (NB-S2)	53,911,567 X2
	30-05-2012 (NB-S3)	68,734,805 X2
	4-06-2012 (NB-S4)	45,344,970 X2
	8-06-2012 (NB-S5)	55,025,398 X2
	Nutrient amendment - control	38,713,917 X2
	-Phosphorus	45,764,698 X2
	+Phosphorus	37,528,376 X2
	-Nitrogen	36,671,310 X2
	+Nitrogen	49,217,942 X2
